# Short-term effects of denervation in the treatment of hypertension: A meta-analysis excluding drug interferences

**DOI:** 10.1097/MD.0000000000040705

**Published:** 2024-11-29

**Authors:** Yimu Wang, Jingyu Liu, Lingyu Wang, Xiang Wang, Huiling Zhang, Haiyan Fang

**Affiliations:** a College of Nursing, Anhui University of Chinese Medicine, Hefei, China.

**Keywords:** antihypertensive agents, catheter ablation, hypertension, meta-analysis, renal denervation

## Abstract

**Background:**

To evaluate the short-term efficacy of denervation in treating hypertension with the exclusion of drug-interfering factors.

**Methods:**

An electronic search was conducted across 8 databases, including MEDLINE, PubMed, Cochrane Library, and EMBASE, for articles on denervation in the treatment of medication-naïve hypertension published from inception to May 2024. All data were meta-analyzed using RevMan 5.3 software.

**Results:**

Four studies, comprising a total of 752 subjects, were screened according to the inclusion and exclusion criteria. Meta-analysis indicated that, compared to the sham-operated group, the denervation group showed a significant reduction in short-term 24-hour ambulatory systolic blood pressure and office systolic blood pressure (OSBP) as well as office diastolic blood pressure (ODBP). No significant safety events were identified.

**Conclusion:**

Denervation has the potential to reduce blood pressure in the short-term for patients with medication-naïve hypertension, demonstrating an acceptable safety profile. This offers hope for patients who are intolerant to drug therapy or unwilling to take lifelong medication. However, its long-term effects require further study. Future research should focus on expanding the sample size and prolonging the follow-up period to further solidify its role in the treatment of hypertension.

## 1. Introduction

Hypertension (HTN) is a global chronic noncommunicable disease that can impair the function and structure of vital organs such as the heart, brain, and kidneys. It significantly contributes to the global burden of disease and economic strain.^[1]^ Compared to individuals with normal blood pressure, those with hypertension are at a higher risk of disease complications.^[2]^ Meta-analysis has shown that for every 5 mm Hg reduction in systolic blood pressure, the risk of major cardiovascular events decreases by 10%. Additionally, the risks of stroke, heart failure, ischemic heart disease, and cardiovascular death are reduced by 13%, 13%, 8%, and 5%, respectively. Lowering blood pressure provides significant health benefits regardless of a patient’s baseline blood pressure and whether or not they have co-morbid cardiovascular disease.^[3]^ Therefore, active blood pressure control is essential to enhance the quality of life and improve the prognosis of hypertensive patients. Currently, lifestyle changes and medication are the cornerstones of HTN treatment. However, many patients are not adequately treated. This issue is especially pronounced in China, where approximately 1.7 million adults have hypertension, and only 7.2% of them achieve successful blood pressure control.^[4]^ In the face of refractory hypertension, low patient compliance, medication intolerance, or resistance to lifelong medication, the search for new therapeutic approaches is particularly urgent.

Renal denervation (RDN) is a minimally invasive hypertension treatment technique that reduces sympathetic nerve activity in the kidneys by ablating sympathetic nerve fibers around the renal arteries, thereby lowering blood pressure.^[1]^ RDN demonstrates potential advantages over conventional treatments in terms of reducing the number of medications used, lowering blood pressure, and improving blood pressure control.^[5]^ Despite initial progress in the clinical use of RDN, its actual contribution to blood pressure reduction, as well as its safety and efficacy in treating HTN, remain controversial due to limitations in study design, the influence of antihypertensive medications, and changes in patient compliance during trials.

Given this context, this study aimed to objectively assess the safety and efficacy of RDN treatment through meta-analysis, excluding interfering factors related to antihypertensive medications, to provide evidence supporting its clinical application. This study was registered in the PROSPERO database (CRD42024537029) to ensure transparency and scientific validity.

## 2. Methods

### 2.1. Search strategy

A comprehensive literature search was conducted using 8 databases: PubMed, EMBASE, Cochrane Library, Web of Science, China National Knowledge Infrastructure (CNKI), WanFang Database, VIP Database, and Chinese Biomedical Literature Database (CBM). The search strategy combined both subject terms and free-text terms. The search timeframe spanned from the establishment of each database to May 2024. The search terms included “hypertension/arterial hypertension/essential hypertension/arterial pressure/blood pressure” “renal denervation/renal sympathetic denervation” “Randomized Controlled Trial/Controlled Clinical Trial.” References from published reviews and meta-analyses were also examined to identify additional eligible studies.

### 2.2. Inclusion and exclusion criteria

Inclusion criteria: Study subjects: Patients with a clear diagnosis of hypertension or those taking antihypertensive medication; age ≥ 18 years; Interventions: Subjects were required to discontinue all antihypertensive medications before the study to establish a stable baseline blood pressure. Participants were then randomized to either the RDN group or the control group (sham operation group); Study outcome indicators: The primary efficacy endpoint was 24-hour ambulatory systolic blood pressure (24h ASBP); secondary efficacy endpoints included 24-hour ambulatory diastolic blood pressure (24h ADBP), office systolic blood pressure (OSBP), and office diastolic blood pressure (ODBP). Safety assessments included all-cause mortality, renal failure, severe hypotension or syncope, hypertensive crisis, and renal artery stenosis; Study type: Randomized controlled trials (RCTs).

Exclusion criteria: Duplicate publications; articles with incomplete or unusable data; lack of full-text availability; literature with serious quality issues; reviews or conference abstracts.

### 2.3. Data extraction

Literature searches were conducted independently by 2 researchers according to the inclusion and exclusion criteria. The literature was de-duplicated and screened using EndNote X9 software, with a third researcher resolving any disagreements. If multiple documents reported on the same study, the document with the most comprehensive information was selected. Two researchers independently extracted data, including author, year of publication, study name, clinical trial registration number, enrollment criteria, sample size, follow-up time, and outcome indicators.

### 2.4. Risk of bias

Quality assessment of included RCTs was performed using the Cochrane Handbook of Risk Assessment version 5.1.^[6]^ The assessment criteria included random sequence generation, allocation concealment, blinding of study conductors and subjects, blinding of outcome assessment, completeness of outcome data, possibility of selective reporting, and other sources of bias. Studies were categorized as “low risk” “unclear” or “high risk” for each criterion. Studies were graded as A if they fully met these requirements, B if they partially met them, and C if they did not meet them and were therefore excluded.

### 2.5. Data analysis

Meta-analysis was performed using RevMan 5.3 software. Heterogeneity was assessed using the chi-square test and the *I*² index. An *I*² < 50% and *P* > .1 indicated acceptable heterogeneity, allowing for a fixed-effects model in the meta-analysis. An *I*² > 50% and *P* < .1 indicated significant heterogeneity, leading to further examination of its sources. After excluding obvious clinical and methodological heterogeneity, a random-effects model was used for meta-analysis. If heterogeneity remained high and could not be explained, descriptive analyses were employed. A *P*-value < .05 was considered statistically significant. Outcome indicators in this study were continuous variables, with effect sizes expressed as mean difference (MD) or standardized mean difference (SMD) and a 95% confidence interval.

## 3. Results

### 3.1. Study selection

**Figure 1.**
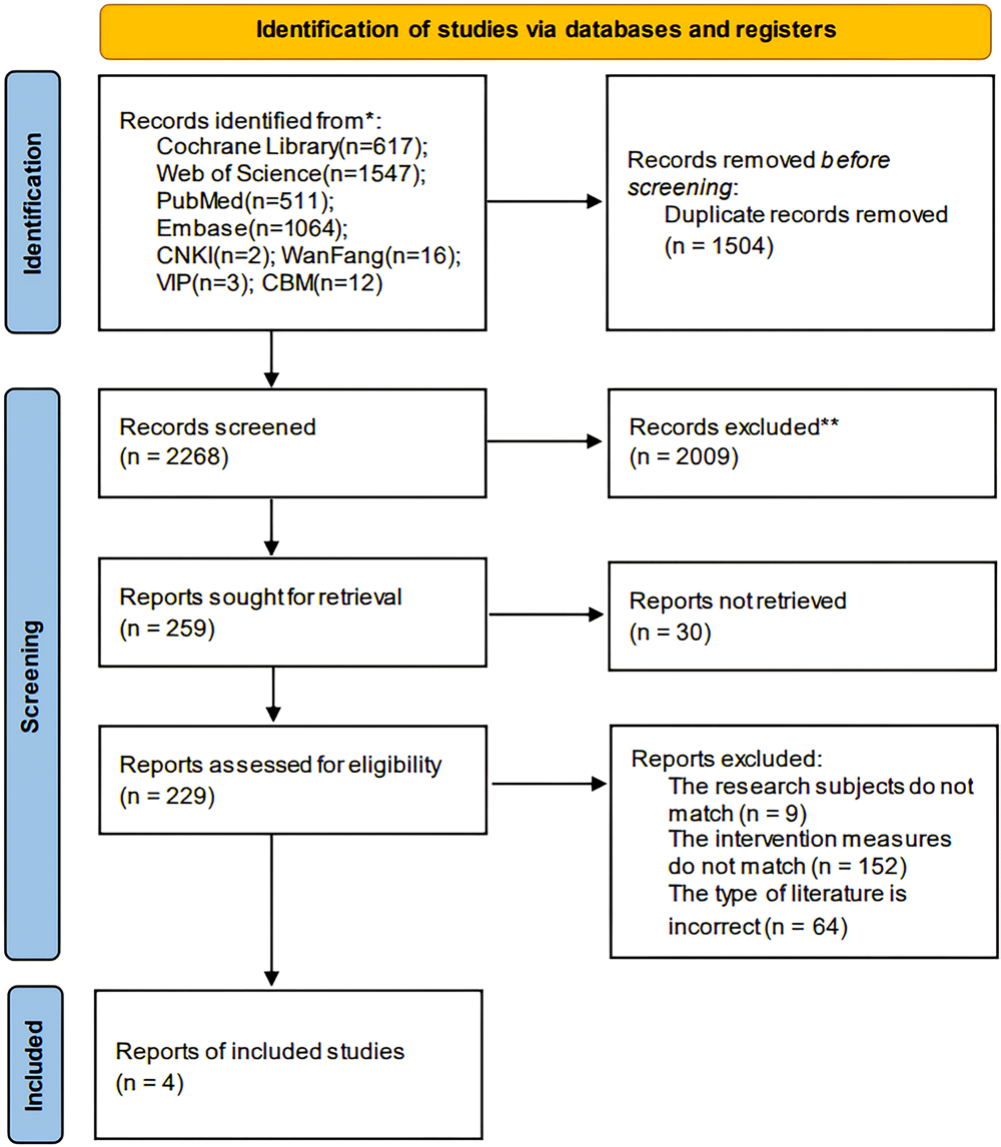
Flow diagram of literature screening.

A total of 3772 articles were retrieved from the literature. After screening using EndNote X9, reading the titles and abstracts, and full-text review, 4 RCTs^[7–10]^ met the inclusion criteria and were included in the analysis. The flow diagram of study retrieval and selection is shown in Figure 1.

### 3.2. Characteristics of included studies

**Figure 2.**
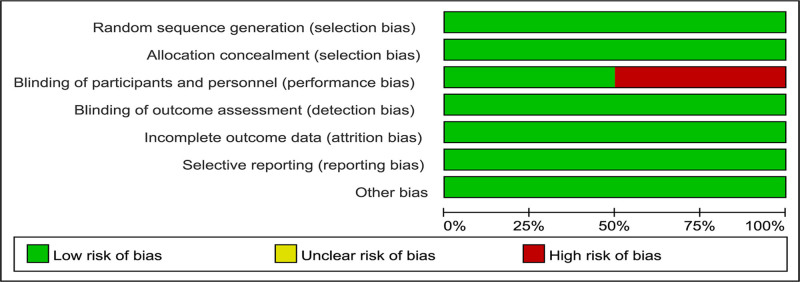
Bias risk assessment of the included studies.

A total of 4 studies, including 752 patients, were included.^[7–10]^ The basic characteristics of these studies are summarized in Table 1. The quality assessment showed that 2 studies^[7,10]^ were graded A and 2^[8,9]^ were graded B. The results of the assessment are shown in Figure 2.

**Table 1:** Characteristics of included studies.

Study	Year	Study title	Clinical trial registration number	Study design	Sample size	Outcomes	Follow-up duration (mo)
Weber^[7]^	2020	REDUCE HTN	NCT02392351	Prospective, randomized, blinded, multicenter study	22/29	(1) (2) (3) (4)	2
Azizi^[8]^	2018	RADIANCE-HTN SOLO	NCT02649426	Multicenter, international, single-blind, randomized, sham-controlled trial	85/61	(1) (2) (3) (4)	2
Böhm^[9]^	2020	SPYRAL HTN-OFF MED Pivotal	NCT02439749	Multicenter, international, single-blind, randomized, sham-controlled, proof-of-concept trial	220/111	(1) (2) (3) (4)	3
Azizi^[10]^	2023	RADIANCE II	NCT03614260	Multicenter, international, single-blind, randomized, sham-controlled trial	160/64	(1) (2) (3) (4)	2

Outcomes: (1) 24-hour ambulatory systolic blood pressure (24h ASBP); (2) 24-hour ambulatory diastolic blood pressure (24h ADBP); (3) Office systolic blood pressure (OSBP); (4) Office diastolic blood pressure (ODBP).

### 3.3. Results of meta-analysis

#### 3.3.1. 24h ASBP

**Figure 3.**
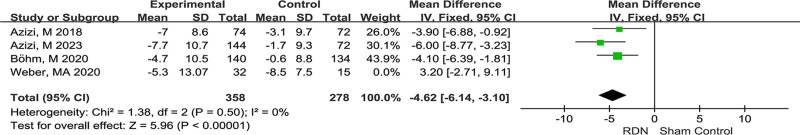
Forest plot of 24h ASBP after removal of heterogeneity. 24h ASBP = 24-hour ambulatory systolic blood pressure.

Four studies^[7–10]^ reported changes in 24h ASBP between the RDN group and the sham-operated group. The results showed significant heterogeneity (*I*² = 61%, *P* = .05). Sensitivity analysis revealed a significant reduction in heterogeneity after excluding the study by Weber^[7]^ (*I*² = 0%, *P* = .50), likely due to the use of different ablation instruments. Meta-analysis using a fixed-effects model after excluding this study showed that RDN treatment significantly reduced 24hASBP by a mean of 4.62 mm Hg compared to the sham surgery group, with a statistically significant difference [MD = −4.62, 95% CI (−6.14, −3.10), *P* < .01] (Fig. 3).

#### 3.3.2. 24h ADBP

**Figure 4.**
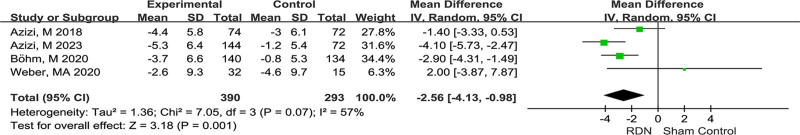
Forest plot of 24h ADBP changes. 24h ADBP = 24-hour ambulatory diastolic blood pressure.

Four studies^[7–10]^ reported changes in 24h ADBP between the RDN group and the sham-operated group. The results showed significant heterogeneity (*I*² = 57%, *P* = .07). The heterogeneity remained high after sensitivity analysis, so a random-effects model was used. The results showed that RDN significantly decreased 24h ADBP by 2.56 mm Hg [MD = −2.56, 95% CI (−4.13, −0.98), *P* < .01] (Fig. 4). Excluding studies one by one revealed that the difference was not statistically significant when the study by Böhm^[9]^ was excluded [MD = −2.07, 95% CI (−4.78, −0.68), *P* = .14].

#### 3.3.3. Office systolic blood pressure

**Figure 5.**
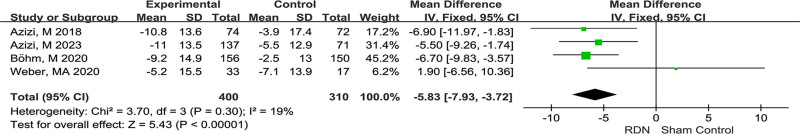
Forest plot of OSBP changes. OSBP = office systolic blood pressure.

Four studies^[7–10]^ reported changes in OSBP between the RDN and sham-operated groups. The results showed no significant heterogeneity (*I*² = 19%, *P* = .30). Using a fixed-effects model, RDN resulted in a statistically significant reduction of 5.83 mm Hg in OSBP [MD = −5.83, 95% CI (−7.93, −3.72), *P* < .01] (Fig. 5). Excluding studies one by one showed that the combined effect sizes were stable and the studies were homogeneous (*I*² < 50%, *P* > .1).

#### 3.3.4. Office diastolic blood pressure

**Figure 6.**
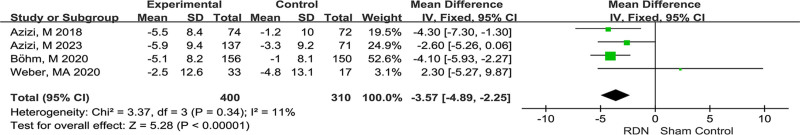
Forest plot of ODBP changes. ODBP = office diastolic blood pressure.

Four studies^[7–10]^ reported changes in ODBP between the RDN and sham-operated groups. The results showed no significant heterogeneity (*I*² = 11%, *P* = .34). Using a fixed-effects model, RDN significantly reduced ODBP by 3.57 mm Hg [MD = −3.57, 95% CI (−4.89, −2.25), *P* < .01] (Fig. 6). Excluding studies one by one showed that the combined effect sizes were stable and the studies were homogeneous (*I*² < 50%, *P* > .1).

### 3.4. Adverse events

Four studies reported on the safety of RDN. Two of these studies reported no adverse events. The study by Weber^[7]^ reported no major device-related or procedure-related safety events within 3 months. However, within 6 months, 1 patient in the RDN group experienced an acute hypertensive crisis that was rapidly reversed with medication. Within 6 to 12 months, 1 patient in the RDN group developed renal artery stenosis (>70%), renal failure, and congestive heart failure, but these symptoms had subsided by the time of presentation. No significant changes in laboratory indices such as estimated glomerular filtration rate (eGFR) or mean blood levels were observed, and there were no significant renal impairments or other hematologic abnormalities during the trial period.

The study by Böhm^[9]^ reported 1 hospital admission for hypertensive emergency in the RDN group and 1 new stroke in the sham-operated group over 3 months. Neither event was caused by the device or procedure. There was no significant difference in safety endpoints between the 2 groups. These findings suggest that RDN has a modest safety profile in the treatment of hypertension, but further attention and evaluation of its potential risks and adverse events are warranted.

### 3.5. Sensitivity analysis and publication bias

Due to the inclusion of fewer than ten papers, funnel plot analysis was not conducted. Sensitivity analysis using a transformed model showed that the fixed-effects and random-effects models produced similar results, indicating stable findings.

## 4. Discussion

The results of this meta-analysis indicate that RDN has a significant short-term effect on lowering 24h ASBP and office blood pressure in hypertensive patients without the use of antihypertensive medications, while demonstrating a favorable safety profile, with no significant safety events directly related to the procedure. These findings are consistent with previous studies,^[7]^ supporting RDN as a potential treatment for hypertension. The advantage of this study over previous ones is the inclusion of patients who were not receiving medication or had discontinued their medication, thus excluding potential confounders such as medication adherence and allowing for a more accurate assessment of the antihypertensive effect of RDN itself.

Blood pressure is one of the risk factors for overall mortality and cardiovascular disease and is usually expressed by systolic and diastolic blood pressure.^[11]^ Elevated blood pressure is strongly associated with an increased risk of cardiovascular disease, and prolonged uncontrolled blood pressure increases the risk of heart disease, stroke, kidney disease, and other conditions.^[12]^ Therefore, regular blood pressure checks and maintenance of healthy blood pressure levels are essential for the prevention of cardiovascular disease. Twenty-four-hour ambulatory blood pressure and office blood pressure are important indicators for evaluating the outcomes of hypertensive patients. Twenty-four-hour ambulatory blood pressure is the preferred technique for measuring blood pressure^[13]^ and improves diagnostic and cardiovascular risk prediction accuracy compared to other methods.^[14]^ Office blood pressure is often used for clinical diagnosis and treatment guidance. Both measurements provide a comprehensive assessment of the impact of RDN on a patient’s blood pressure level and an accurate assessment of treatment efficacy. This findings support RDN as an effective treatment for hypertension, significantly reducing 24h ASBP, OSBP, and ODBP, which may help reduce patients’ risk of cardiovascular disease. This makes RDN a potential alternative for patients unable to achieve desired blood pressure control with conventional pharmacologic therapy. However, due to the presence of heterogeneity and the results of sensitivity analysis, the antihypertensive effect on 24h ADBP in hypertensive patients requires further verification.

Although traditional drug therapy can control blood pressure in some hypertensive patients, its obvious side effects, poor adherence, and poor long-term efficacy limit its clinical application.^[15,16]^ RDN reduces blood pressure by destroying or blocking the renal sympathetic nervous system, providing a long-lasting method of lowering blood pressure independent of drug pharmacokinetics.^[3]^ Compared with drug therapy, RDN reduces the number of necessary antihypertensive medications,^[17]^ thereby improving patient compliance.^[18]^ Additionally, RDN may have a protective effect on the cardiovascular system. For instance, a study by Hanssen^[19]^ noted that RDN significantly reduced left ventricular hypertrophy and improved cardiac diastolic function, which had a positive prognostic impact on patients with refractory hypertension. Delacroix^[20]^ further revealed potential benefits of RDN in terms of improved myocardial perfusion and cardiac function. Moreover, patients with refractory hypertension not only benefited in terms of blood pressure control after RDN treatment but also experienced improvements in mental health and quality of life.^[21]^ These results are consistent with predictions using a state-transition model, which showed that RDN significantly reduced the probability of cardiovascular events such as stroke, myocardial infarction, coronary artery disease, heart failure, and end-stage renal disease, as well as prolonged patient survival and demonstrated cost-effectiveness in terms of economic evaluation.^[22]^ With an increasing number of clinical studies supporting its effectiveness, RDN treatment may become an important therapeutic option for managing hypertension.

However, despite its short-term benefits, the long-term effects and potential challenges of RDN need further exploration. For example, a study by Booth^[23]^ in a sheep model noted that renal sympathetic nerve activity may return to normal 11 months after RDN surgery. Similarly, Bergland^[24]^ found in a long-term follow-up study of up to 7 years that there was no significant difference in long-term blood pressure control between RDN and pharmacological treatments. These findings suggest that the long-term antihypertensive effects of RDN may require reassessment and adjustment of treatment regimens. Future studies should further explore the long-term effects of RDN treatment and potential side effects to more comprehensively assess its clinical prospects.

## 5. Study limitations

Despite our efforts, this study has a few limitations that should be acknowledged:

Only 4 papers were included, limiting the possibility of performing funnel plot analysis to assess publication bias; Due to the study’s methodology, it was not possible to keep patients off medication for a long period to assess the long-term effects of RDN.

Given the results of this study, future research should focus on: Conducting longer follow-up studies to assess the long-term antihypertensive effects and safety of RDN; Conducting large-sample, multicenter clinical trials to enhance the generalizability and strength of the findings; Exploring the effects of different ablation devices and surgical procedures on therapeutic outcomes, as well as differences in efficacy among populations of different races and regions, to optimize the application of RDN.

## 6. Conclusion

In summary, the results of this study indicate that RDN has the potential to reduce blood pressure in hypertensive patients who have not received pharmacological treatment in the short-term and demonstrates acceptable safety. Although more research is needed to determine its long-term effects, RDN offers hope as a new treatment option for patients who are intolerant to medication or unwilling to undergo lifelong drug therapy. Future studies should focus on increasing sample sizes, extending follow-up periods, and evaluating the effects of RDN in diverse racial and regional populations to further solidify its role in hypertension management.

## Author contributions

**Conceptualization:** Yimu Wang.

**Data curation:** Jingyu Liu, Lingyu Wang.

**Formal analysis:** Jingyu Liu, Lingyu Wang.

**Funding acquisition:** Xiang Wang, Huiling Zhang, Haiyan Fang.

**Methodology:** Yimu Wang, Jingyu Liu, Lingyu Wang.

**Project administration:** Yimu Wang, Xiang Wang, Haiyan Fang.

**Software:** Jingyu Liu, Lingyu Wang.

**Supervision:** Xiang Wang, Huiling Zhang, Haiyan Fang.

**Writing – original draft:** Yimu Wang.

**Writing – review & editing:** Xiang Wang, Huiling Zhang, Haiyan Fang.
